# Delayed onset of *Corynebacterium simulans* infection following open reduction and internal fixation of a trimalleolar fracture

**DOI:** 10.1093/jscr/rjae334

**Published:** 2024-05-26

**Authors:** Konstantinos Tsivelekas, Stavros Lykos, Dimitrios Pallis, Margarita-Michaela Ampadiotaki, Petros Nikolakakos, Anastasia Tilentzoglou, Stamatios A Papadakis

**Affiliations:** B' Department of Orthopaedics, KAT General Hospital of Athens, Nikis 2 str. 15124, Greece; B' Department of Orthopaedics, KAT General Hospital of Athens, Nikis 2 str. 15124, Greece; B' Department of Orthopaedics, KAT General Hospital of Athens, Nikis 2 str. 15124, Greece; B' Department of Orthopaedics, KAT General Hospital of Athens, Nikis 2 str. 15124, Greece; B' Department of Orthopaedics, KAT General Hospital of Athens, Nikis 2 str. 15124, Greece; B' Department of Orthopaedics, KAT General Hospital of Athens, Nikis 2 str. 15124, Greece; B' Department of Orthopaedics, KAT General Hospital of Athens, Nikis 2 str. 15124, Greece

**Keywords:** surgical site infection, ankle fracture, Corynebacterium, C. Simulans, ORIF

## Abstract

Surgical site infections (SSIs) following open reduction and internal fixation (ORIF) of ankle fractures can lead to significant disability. This case report emphasizes a unique instance of SSI caused by *Corynebacterium simulans*, following ORIF of a trimalleolar ankle fracture in a 55-year-old female patient. To our knowledge, this is the first reported case of *C. simulans* infection after ORIF in the literature. The pathogen was detected after surgical debridement, removal and sonication of the hardware, and identified through matrix assisted laser desorption/ionization-time of flight mass spectrometry (MALDI-TOF MS) and 16S rRNA gene sequencing. Specific intravenous antibiotic regimen was administered for a total duration of 4 weeks. During the 12th month follow-up, the patient presented no signs of infection and an excellent clinical outcome. This case report underscores the need for alertness regarding atypical pathogens in postoperative complications and the critical role of precise microbial diagnosis in managing rare orthopaedic infections.

## Introduction

Ankle fractures constitute one of the most common types of fractures in orthopaedic surgery, accounting for almost 8% of all fractures in adults and more than 30% of all tibia and fibula fractures. Among the elderly population, ankle fractures are the third most common type of fracture, preceded only by hip and wrist fractures [1]. Almost 50% of ankle fractures require surgical intervention, with open reduction and internal fixation (ORIF) being the predominant treatment option [2].

Despite the precautions adopted, postoperative surgical site infections (SSIs) are reported at varying rates, ranging from 1% to 26% [3]. Given the gradual and usually silent process of infection and the formation of biofilm, both diagnosis and treatment can be difficult [4]. Hence, SSI constitutes one of the most significant complications, potentially leading to permanent or severe disability and amputation in most severe cases. Moreover, SSI contribute to increased hospitalization costs due to prolonged hospital stays, additional surgical interventions, extended rehabilitation periods, and augmented use of antibiotics [2].

Currently, *Pseudomonas aeruginosa*, methicillin-resistant *Staphylococcus aureus*, and methicillin-susceptible *S. aureus* are the most frequent responsible pathogens in orthopaedic surgery [1]. Ankle fractures SSI involving *Corynebacterium* species are rare. The literature records only a few instances of infections caused by this genus, while particularly the *C. simulans* species are exceptionally uncommon in orthopaedic surgery [4, 5].

This study reports an extremely rare case, likely the first ever reported in the literature, of a SSI caused by *Corynebacterium simulans*, following ORIF for a trimalleolar fracture in a healthy female patient. The objective of this report is to provide clinicians with critical information for the effective selection of antibiotics and treatment approaches in managing bone and soft tissue infections attributed to the *Corynebacterium* genus, emphasizing the unique and scarce nature of such cases.

## Case presentation

**Figure 1:**
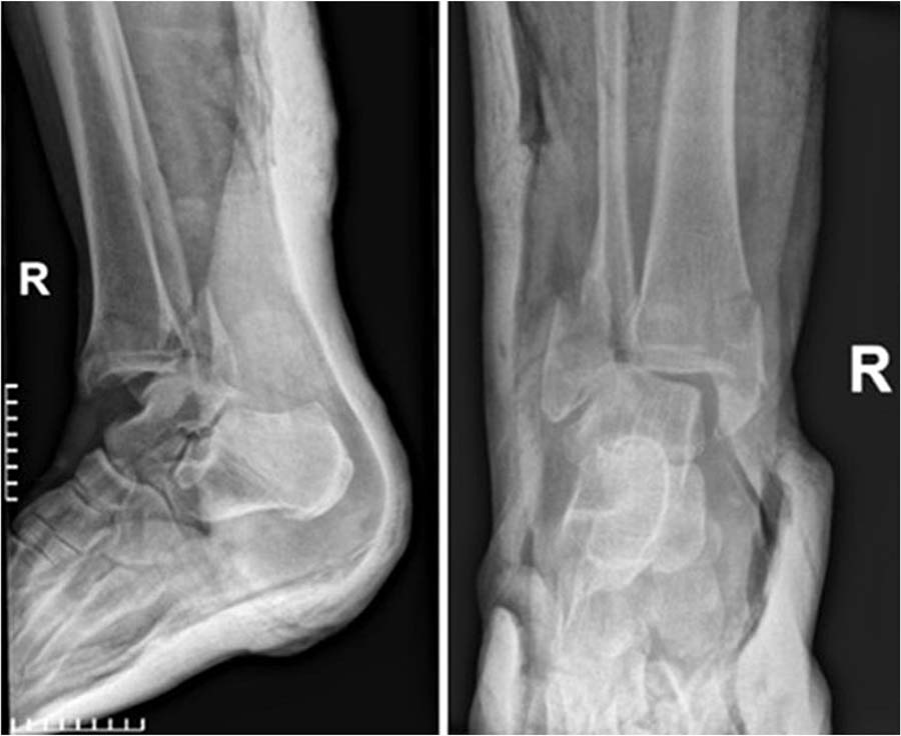
Anteroposterior and lateral X-ray of the ankle joint showing the trimalleolar fracture.

A 55-year-old female patient was admitted to the emergency department of our hospital due to an inability of weight bearing following a fall from standing height. Her medical history was unremarkable, with no significant records or regular medication use. Radiological evaluation revealed a trimalleolar fracture of the right ankle. Consequently, a cast was applied for pain management, and no weight-bearing was allowed on the affected limb (Fig. 1).

**Figure 2:**
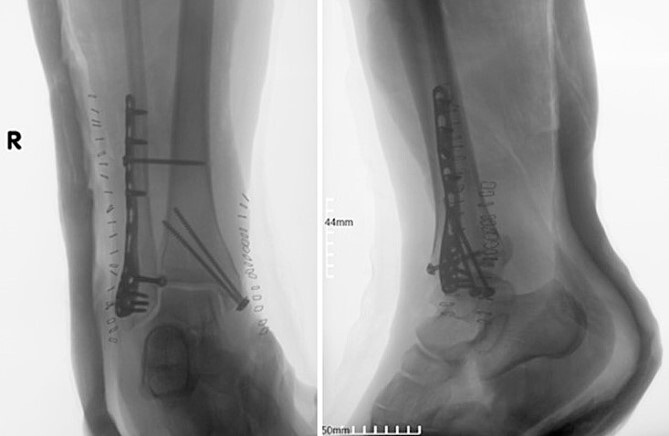
Postoperative anteroposterior and lateral X-ray of the ankle joint after ORIF of the fracture.

A surgical treatment was decided, consisting of ORIF of the fracture which was addressed within 48 hours of the admission to the hospital. Fracture reduction was achieved through a lateral and medial approach to the lateral and medial malleolar, respectively. A 3.5-mm neutralization plate with a lag screw was applied on the lateral malleolus combined with a 3.5-mm lag screw fixation of the medial malleolus and a 3.5-mm syndesmotic screw fixation (Fig. 2).

**Figure 3:**
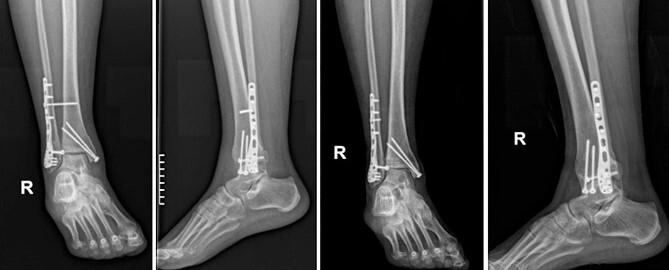
Anteroposterior and lateral X-ray of the ankle joint showing the broken syndesmotic screw and the replacement with a 3.5-mm cortical screw on the lateral malleolus 8 weeks after the initial surgery.

Four doses of i.v. cefoxitin (2000 mg 5 minutes prior to skin incision in the operating room and 1000 mg three times postoperatively every 8 h) were administrated as chemoprophylaxis. The postoperative period was well-progressed, and the patient was discharged on the second postoperative day with a cast and instructions against weight-bearing. In the seventh postoperative week, the patient deviated from the prescribed postoperative regimen by prematurely initiating weight-bearing activities, contrary to the surgeon’s explicit recommendations. During the regular 8-week follow-up, we observed after the radiological evaluation that the syndesmotic screw was broken. This necessitated us to remove the screw and replace it with a 3.5-mm cortical screw (Fig. 3).

The patient received the same protocol of chemoprophylaxis and was discharged on the first postoperative day. Partial and gradually increased weight bearing was suggested, until fully weight bearing on the second postoperative week.

During the 6-month regular follow-up period, the patient reported discomfort and pain while walking and had an American Orthopaedic Foot and Ankle Society (AOFAS) score of 76. At this time, blood tests were negative for infection accounting: white blood cells (WBC) = 7,6 × 10^3^/μl (normal values = 4.6–10.2 × 10^3^/μl), C-reactive protein (CRP) < 0.31 mg/dl (normal values <0,31 mg/dl), and erythrocyte sedimentation rate (ESR) = 14 mm/h (normal values = 0–20 mm/h). A decision was made to remove the hardware, however, due to the patient’s COVID infection the intervention was postponed. Eight months postoperatively, hardware was removed due to surgical site pain, exacerbated by the patient’s inability to tolerate the discomfort at the surgery site.

The patient received the same antibiotics for chemoprophylaxis. During the procedure, surgical debridement was performed. Six specimens of soft tissue cultures were obtained during the hardware removal, and a sonication of the plate and the screws was conducted by the Department of Microbiology of our hospital.

*Corynebacterium simulans* was accurately identified in the Department of Microbiology of our hospital. Primarily, the organism was detected through matrix-assisted laser desorption/ ionization-time of flight mass spectrometry (MALDI-TOF MS) via the VITEK MS system. Further confirmation of these results was ensured by 16S rRNA gene sequencing. Additionally, PCR testing using specific primers, such as ssrA-Fwd and ssrA-Rev, also yielded positive amplification for *C. simulans*.

*Corynebacterium simulans* was identified in all soft tissue specimen. Hardware sonication revealed *C. simulans* spp. isolation from the plate and screws of the lateral malleolus, while the screws of the medial malleolus did not reveal any pathogen. Following the identification of the *Corynebacterium* spp. and the antibiotic susceptibility testing (Table 1), the patient was subsequently treated with a rigorous antibiotic regimen. This included an initial dose of 1000 mg of intravenous vancomycin twice daily. Due to suboptimal blood levels of vancomycin (recorded at 4.5mcg/ml after 4 days), the dosage was increased to 1000 mg three times per day. During the third week of treatment, the patient received two intravenous doses of 500 mg dalbavancin twice on a single day. A follow-up dose of 500 mg was administered a week later. The total duration of i.v. antibiotic medication was 4 weeks. Both CRP and ESR were evaluated twice per week. CRP levels normalized just 1 week after beginning treatment. Post-treatment, the patient reported no pain at the site of infection and gradual weight bearing was recommended.

**Table 1:** Susceptibility testing of the presented case.

	1			1	
	Resistance	MIC		Resistance	MIC
Clindamycin	R		Penicillin	S	0,064
Gentamycin	S	0,016	Rifampicin	S	0,016
Linezolid	S	0,25	Vancomycin	S	0,75

S = susceptible, I = intermediate, R = resistant, MIC = minimum inhibitory concentration mcg/ml.

During the 12th month follow-up, the CRP and ESR yielded results within normal ranges and indicated no signs of ongoing infection. The patient had an AOFAS score of 87.

## Discussion

SSI following ankle fractures ORIF consist of a significant complication requiring both immediate diagnosis and optimal management approach to avoid devasting consequences. To date, gram-positive cocci, particularly *S. aureus* and coagulase-negative *Staphylococci* species have been the most commonly identified and well-studied pathogens in orthopaedic surgery, remarking significant rates of SSI and prosthetic joint infections (PJIs) [6].

*Corynebacterium* species are relative rare in orthopaedic surgery, with the vast majority of them concerning *Corynebacterium striatum* spp. [5, 7–9]. However, few authors have reported that *Corynebacterium* spp. have been neglected among orthopaedic infections since they represent almost 3% of the responsible pathogens in PJIs [10, 11]. Besides that, almost all authors consent and highlight that *Corynebacterium* spp. are truly difficult to detect, and the management of such patients remains challenging [5, 7, 9–12]. Our study presents the first documented case in the literature of a SSI caused by *C. simulans* in a 55-year-old female patient, following ORIF. This case adds to the limited body of knowledge on this organism, particularly in the context of SSI in the orthopaedic field [5, 9].

*Corynebacterium* species are Gram-positive, slender, non-spore-forming rod-like structures that exhibit slight curvatures or club-shaped ends. These organisms typically measure 0.3–0.8 μm in diameter and 1.5–8.0 μm in length. Facultatively anaerobic and catalase-positive, *Corynebacterium* are generally nonmotile, functioning under facultatively anaerobic conditions through fermentation [13]. Pervasively distributed in diverse environments, they can be isolated from soil, water, plant material, and animals, with a current classification including 88 species and 11 subspecies. *C. simulans*, identified in 2000 by Wattiau *et al*., primarily inhabits the skin and has rare potential to cause infections [14]. Its genome shares significant similarity with the nucleotide sequences of *C. striatum* (98.0%) and *C. minutissimum* (96.9%) both noted for their high antibiotic resistance. Identifying *C. simulans* in routine clinical practice poses substantial challenges due to the complexity of the necessary tests [14, 15]. It is important to note that routine clinical testing methods, like VITEK 2, API Coryne, and RapID CB Plus, often misclassify *C. simulans* due to its absence in conventional test databases. Therefore, there is reliance on more sophisticated methods, with MALDI-TOF MS and genetic sequencing being used for accurate identification [13, 16].

To our knowledge, our study signifies the first SSI caused by *C. simulans* after internal fixation of a fracture in a healthy patient, where pain and discomforts were the only sign of the infection. Previously, Ogasawara *et al*. had described a case of acute pyogenic spondylitis caused by *C. simulans* in a 78-year-old man with diabetes mellitus [16]. Additionally, few reports have pointed out several cases of native osteomyelitis and PJIs caused by *C. simulans* [5, 7–11]. However, in most cases of SSI, a well-established association with various predisposing factors and comorbidities has been implicated [1, 2, 16].

The rarity of *C. simulans* has been emphasized by Ogasawara *et al*. in their retrospective observational study in 2021, providing valuable insights into the pathogen. The authors analyzed 13 strains of *C. simulans* and 317 strains of *C. striatum*, focusing on patient background, specimen types, and antimicrobial susceptibilities. The study revealed that *C. simulans* accounted for only 3.9% of isolations compared to 96% for *C. striatum*. Interestingly, *C. simulans* was predominantly found in skin specimens (61.5%) and rarely in mucous membrane-associated specimens such as sputum or craniocervical secretions, where *C. striatum* was more frequently detected [13]. This finding is particularly relevant to our case, where the infection was associated with a surgical site on the skin.

As far as the antimicrobial susceptibility is concerned, *C. simulans* has shown a higher susceptibility to several antibiotics compared to *C. striatum*. Notably, *C. simulans* seems to be more susceptible to penicillin G, ceftriaxone, and ciprofloxacin [9, 17]. Corresponding to our treatment approach, the patient responded well to vancomycin and dalbavancin, emphasizing the effectiveness of certain antibiotics against this pathogen. *Corynebacterium* infections have been reported to be most susceptible against linezolid and vancomycin, as well as daptomycin even in multiresistant cases [4, 18, 19]. Also, Chauvelot *et al*. [4] reported significant susceptibility to tetracycline. The development of new antibiotics against gram-positive pathogens showed fair outcomes in the treatment of soft tissue and skin infections including oritavancin, omadacycline, dalbavancin, tedizolid, telavancin, eravacycline, and delafloxacin [20]. The availability and documentation in the literature regarding the use of newer drugs for treating *Corynebacterium* infections remain limited, owing to the scarcity of clinical experience and the absence of data concerning their in vivo efficacy. Typically, i.v. administrations are mandatory against *Corynebacterium* spp. due to their great resistance to per os antibiotics [18].

## Conflict of interest statement

The authors declare that there is no actual or potential conflict of interest in relation to this article.

## Funding

All authors have declared that no financial support was received, and they have no financial relationships at present or within the previous years with any organization that might have an interest in the submitted work.

## Disclosures

Written informed consent was obtained by the patient to participate in this study. The study was approved by the Scientific Committee of our hospital (SC approval no: 72307).
